# Cell-Associated Flagella Enhance the Protection Conferred by Mucosally-Administered Attenuated *Salmonella* Paratyphi A Vaccines

**DOI:** 10.1371/journal.pntd.0001373

**Published:** 2011-11-01

**Authors:** Orit Gat, James E. Galen, Sharon Tennant, Raphael Simon, William C. Blackwelder, David J. Silverman, Marcela F. Pasetti, Myron M. Levine

**Affiliations:** 1 Center for Vaccine Development, University of Maryland School of Medicine, Baltimore, Maryland, United States of America; 2 Department of Medicine, University of Maryland School of Medicine, Baltimore, Maryland, United States of America; 3 Department of Microbiology and Immunology, University of Maryland School of Medicine, Baltimore, Maryland, United States of America; 4 Department of Pediatrics, University of Maryland School of Medicine, Baltimore, Maryland, United States of America; Massachusetts General Hospital, United States of America

## Abstract

**Background:**

Antibiotic-resistant *Salmonella enterica* serovar Paratyphi A, the agent of paratyphoid A fever, poses an emerging public health dilemma in endemic areas of Asia and among travelers, as there is no licensed vaccine. Integral to our efforts to develop a *S.* Paratyphi A vaccine, we addressed the role of flagella as a potential protective antigen by comparing cell-associated flagella with exported flagellin subunits expressed by attenuated strains.

**Methodology:**

*S.* Paratyphi A strain ATCC 9150 was first deleted for the chromosomal *guaBA* locus, creating CVD 1901. Further chromosomal deletions in *fliD* (CVD 1901D) or *flgK* (CVD 1901K) were then engineered, resulting in the export of unpolymerized FliC, without impairing its overall expression. The virulence of the resulting isogenic strains was examined using a novel mouse LD_50_ model to accommodate the human-host restricted *S.* Paratyphi A. The immunogenicity of the attenuated strains was then tested using a mouse intranasal model, followed by intraperitoneal challenge with wildtype ATCC 9150.

**Results:**

Mucosal (intranasal) immunization of mice with strain CVD 1901 expressing cell-associated flagella conferred superior protection (vaccine efficacy [VE], 90%) against a lethal intraperitoneal challenge, compared with the flagellin monomer-exporting mutants CVD 1901K (30% VE) or CVD 1901D (47% VE). The superior protection induced by CVD 1901 with its cell-attached flagella was associated with an increased IgG2a∶IgG1 ratio of FliC-specific antibodies with enhanced opsonophagocytic capacity.

**Conclusions:**

Our results clearly suggest that enhanced anti-FliC antibody-mediated clearance of *S.* Paratyphi A by phagocytic cells, induced by vaccines expressing cell-associated rather than exported FliC, might be contributing to the vaccine-induced protection from *S.* Paratyphi A challenge *in vivo*. We speculate that an excess of IgG1 anti-FliC antibodies induced by the exported FliC may compete with the IgG2a subtype and block binding to specific phagocyte Fc receptors that are critical for clearing an *S.* Paratyphi A infection.

## Author Summary

*Salmonella enterica* serovar Paratyphi A is a pathogen that causes a systemic disease that is marked by serious complications and, if untreated, high mortality. The study of *S.* Paratyphi A pathogenesis and vaccine development has been extremely challenging since *S.* Paratyphi A is human host-restricted and no appropriate animal model exists. Since there is currently no licensed vaccine to prevent paratyphoid fever caused by this organism, our study represents a pioneering attempt to develop and refine a vaccine against *S.* Paratyphi A. We employed live attenuated strains which allow *in vivo* presentation of bacterial antigens via the natural route of infection, without the complications associated with antigen production and purification for subunit vaccines. For determining protective immunity against infection, we developed a mouse model that allowed evaluation of vaccine efficacy. We used our system to examine the protective capacity of a major *Salmonella* antigen, the flagellum. Due to its unique immunogenic properties, the flagellum is considered a major immune mediator, but its role in protection is controversial. We clearly show that cell-associated flagellar protein, presented by mucosally administered attenuated bacterial live vaccines, provides superior protection when compared to strains exporting FliC monomers, and we discuss possible mechanisms of immunity.

## Introduction

Four human host-restricted *Salmonella enterica* serovars cause clinically indistinguishable typhoid (*Salmonella* Typhi) and paratyphoid (*S.* Paratyphi A, B and [uncommonly] C) fever [1]. Multiply antibiotic resistant *S.* Paratyphi A have emerged in Asia, accompanied by increased incidences of paratyphoid fever in endemic populations [2], [3] and in travelers [4]. Whereas vaccines exist to prevent typhoid fever, there is no licensed vaccine to prevent *S.* Paratyphi A disease. Vaccines for preventing typhoid fever include the purified Vi capsular polysaccharide administered parenterally and attenuated Vi-negative strain Ty21a given orally as a live vaccine [5]. Vi conjugated to recombinant exotoxin A of *Pseudomonas aeruginosa* conferred on Vietnamese children a high level of efficacy in a field trial [6], [7]. Vi-based vaccines cannot protect against paratyphoid disease as *S.* Paratyphi A does not express Vi. Oral Ty21a confers moderate cross protection against *S.* Paratyphi B [8] but not *S.* Paratyphi A [9]. Despite the public health need [4], there have been few reports on modern *S.* Paratyphi A vaccine development [10], [11].

Attenuated *Salmonella* strains can be employed as mucosally-delivered vaccines or as “reagent strains” to achieve safe, high-yield production of purified antigens for manufacture of parenteral (conjugate) vaccines. A *Salmonella* surface antigen that has generated renewed interest in the role that it may play in protection is the flagellum. Flagella mediate intestinal epithelial and macrophage inflammation following infection and contribute to early host innate immune responses against *Salmonella* [12]. Flagellin (FliC), the monomer of flagellar filaments that induces these effects, is being incorporated into fusion proteins linked to otherwise poorly immunogenic antigens and haptens, providing adjuvant activity to enhance immune responses to those moieties [13]–[15].

The flagellum is a complex motility organelle composed of >20 different proteins that form a basal body, hook, filament and an export system. The major extracellular part of the flagellum comprises ∼20,000 FliC monomers that are exported and assembled at the terminus of a growing filament. Between the hook and filament is a short junction formed by two hook-associated proteins, FlgK and FlgL [16], [17]. *S.* Typhimurium mutants defective in FlgK or FlgL synthesize FliC monomers that do not polymerize and are released into the culture medium [17]. A capping structure of five FliD molecules at the end of the filament also promotes FliC polymerization [16], [18], [19]. Deletion of *fliD* in *S.* Typhimurium incapacitates the ability of transported FliC to polymerize [20], [21].

Integral to our efforts to develop a *S.* Paratyphi A vaccine, we addressed the role of flagella as a potential protective antigen by comparing cell-associated flagella with exported flagellin subunits expressed by attenuated strains. Mutants were constructed with deletions in *fliD* or *flgK*, resulting in export of unpolymerized FliC, without impairing its overall expression. These strains allowed us to investigate whether expression of FliC as cell-attached flagellin filaments versus exported monomers, would influence the immune response or protection elicited by these live vaccines.

## Materials and Methods

### Ethics statement

All animal experiments carried out in this work were approved by the University of Maryland Baltimore Office of Animal Welfare Assurance (OAWA), under approved Animal Use Protocol 0409006.

### Bacterial strains, media and growth conditions

*S.* Paratyphi A wild-type and mutant strains (Table 1) were propagated on animal product-free LB Lennox medium (Athena ES, Baltimore, MD). Lennox agar plates were prepared by addition of 1.5% agar (Difco, BD, Franklin Lakes, NJ). Guanine (0.001% v/v) was added for Δ*guaBA* mutant strains. Liquid cultures were incubated at 37°C, 250 rpm, at a ratio of 1∶10–1∶20 vol∶vol medium∶flask (high-aeration conditions). For low-aeration growth conditions, the flasks were filled to 75% of their volume with the medium, and shaken at 80 rpm. Time course experiments in liquid culture were seeded with an overnight culture, inoculated to 0.01 OD_600_; samples were removed at regular intervals for determining culture turbidity at OD_600_ or plating. For each growth experiment, two flasks were cultured per strain, and each experiment was performed twice. Swimming and swarming assays were carried out on fresh Lennox plates containing 0.3% and 0.5–0.7% agar, respectively. Glucose was added to a final concentration of 0.5% for swarm plates. The swim plates were inoculated by stabbing the center with bacteria harvested from 1.5% Lennox plates. The swarm plates were inoculated by pipetting a 10-µl fresh culture, grown in liquid Lennox media to 0.5–0.6 OD_600_, onto the surface of the center of the agar plate. All motility experiments were performed in triplicates and repeated at least twice. Swim and swarm ability are expressed as the radius of the mobility zone; for no swim, the radius of growth was >1 mm and for no swam, 10 mm.

**Table 1:** *Salmonella* Paratyphi A strains and their relevant characteristics.

Strain	description	Source
ATCC 9150	WT	CVD-Baltimore collection
Q28b	Clinical isolate	CVD-Baltimore collection, isolated in Mali
EAR 6473	Clinical isolate	CVD-Baltimore collection, isolated in Chile
15.067	Clinical isolate	CVD-Baltimore collection, isolated in Chile
CVD 1901	ATCC 9150 Δ*guaBA*	Levine MM, unpublished
CVD 1902	CVD 1901 Δ*clpX*	Levine MM, unpublished
9150*D*	ATCC 9150 Δ*fliD*	This study
9150*K*	ATCC 9150 Δ*flgK*	This study
CVD1901*D*	CVD 1901 Δ*fliD*	This study
CVD1901*K*	CVD 1901 Δ*flgK*	This study

### Mutagenesis

Deletion of *fliD* and *flgK* genes was performed by λ Red-mediated mutagenesis [22] essentially as described [23]. Primers (listed in Table 2) were designed to replace most of the gene of interest with a kanamycin resistance cassette flanked by the Flippase Recombination Targets, FRTs. The kanamycin cassette was later deleted via λ Red recombinase, leaving an FRT scar sequence.

**Table 2:** Mutagenesis oligonucleotides sequence.

Designation	Use*	Sequence (5′ to 3′)**
PCR1-5FRT-aph	Km^R^ cassette	gaattcgctagcGCTGGAGCTGCTTCGAAGTTC
PCR1-3FRT-aph	Km^R^ cassette	ctcgagTTCCGGGGATCCGTCGACCTGCAGTTC
PCR2-5FliD	*fliD* deletion	gaattcTCACGCACACGCTGCAGG
PCR2-5FliD-rev	*fliD* deletion	gctagcACCTAATGATGAAATTGAAGCCATGC
PCR3-3FliD	*fliD* deletion	gaattcGCTATGAACAAGTCCTGATAACAGAGGT
PCR3-3FliD-rev	*fliD* deletion	ctcgagTTAACGAGAACTCCTGGAAAGATGCTTTC GGTGAAATCTGC
PCR4-5FlgJ	*flgK* deletion	gaattcGGCGAACCCAGCTATAACGTATTTGGCG
PCR4-3FlgJ	*flgK* deletion	gctagcATTAATCAAGCTGGACATGATGGTTCC
PCR5-5FlgL	*flgK* deletion	agatctGCGTTACTGAATATTCGCTAAAGGAGAAG
PCR5-3FlgL	*flgK* deletion	ctcgagCGTATGGCCAATTACCATCGTGCGTGCG

*Km^R^, Kanamycin resistance gene.

**Restriction endonuclease cleavage sites are underlined. For PCR1, *EcoRI*, *NheI* and *XhoI*; PCR2 and PCR 4, *EcoRI* and *NheI*; PCR3, *BamHI* and *XhoI*; PCR5, *BglII* and *XhoI*.

### SDS-PAGE and western blot analyses

Bacterial protein samples were normalized as follows. Cell pellets were washed in 0.125 M Tris-HCl, pH 6.8, brought to 10 OD_600_ in the same buffer and diluted 1∶3 with Laemmli sample buffer (Bio-Rad Laboratories, Hercules, CA). Supernatants were brought to the equivalent lowest OD_600_ culture per experiment, by addition of 0.125 M Tris-HCl, pH 6.8, and diluted 1∶1 with Laemmli buffer. The protein samples were boiled for 10 min, and 10-µl aliquots were loaded onto 10% SDS-PAGE gels. For anti-FliC blots, monoclonal antibodies (BioVeris, Gaithersburg, MD) diluted 1∶1000 were used for 1 h incubation. Detection was performed with secondary peroxidase-labeled goat anti-mouse IgG (KPL, Gaithersburg, MD), followed by application of the ECL PLUS Western blotting detection system (GE Healthcare, Buckinghamshire, UK). Coomassie blue-stained gels and developed blots were scanned with a V700 Photo EPSON Scan (digital ICE technologies) using SilverFast SE imaging software (LaserSoft Imaging, Sarasota, FL), and quantitated with QuantityOne software (Bio-Rad).

### Preparation of *S.* Paratyphi A flagella protein standard

*S.* Paratyphi A FliC was prepared from strain CVD 1902 using the shearing. CVD 1902 was chosen for purification of flagella for two reasons. First, it was genetically engineered from the attenuated strain CVD 1901 to hyper-express flagellin by deletion of the *clpX* gene (Table 1), which together with *clpP* encodes the ClpXP ATP-dependent protease that degrades the master flagella positive regulator complex FlhD/FlhC, resulting in large amounts of flagella being over-produced [24]. Second, it is an attenuated strain and as such does not pose an occupational risk when cultured in large volumes for antigen purification.

Bacterial cultures were grown overnight under low aeration conditions in 2-liter flasks containing Lennox broth supplemented with guanine. Cell pellets were washed and resuspended in PBS, and sheared for 3 min at high speed in a Waring laboratory blender. The sheared suspension was centrifuged twice at 7,000×*g* for 10 min, and the supernatant was collected and centrifuged at 100,000×*g* for 3 h to pellet the filaments. The pellet was suspended in saline at 4°C overnight, centrifuged at 7,000×*g*, and the clear supernatant containing flagellar filaments was transferred to a new tube. Protein concentration was determined with the BCA assay (Pierce, Rockford, IL). Purity was assessed by SDS-PAGE and Coomassie blue staining. The amount of contaminating LPS was quantified using the resorcinol sulfuric acid assay [25] using a standard curve generated with purified *S.* Paratyphi A LPS. This FliC preparation was determined to be 98.3% pure.

### Ultrafiltration

UF membranes with different *Mr* cut-offs were used for gradient separation of flagellin monomers from purified filaments. Supernatants collected from bacterial cultures grown under high-aeration conditions and containing flagellin monomers and/or sheared flagella, were first normalized to equal concentrations of FliC by passing supernatants through a 30-kDa cut-off Amicon membrane (Millipore, Billerica, MA). The concentrated retentants were then passed through a 100-kDa cut-off Amicon membrane. The resulting filtrates were further passed through an additional 30-kDa membrane.

### Transmission electron microscopy

Bacteria collected from swarm colonies were suspended in PBS to an OD_600_ of 1.0 and were incubated with 300 mesh Formvar coated copper grids (Electron Microscopy Services, Hatfield, PA) for 20 min. Grids were gently blotted and placed on 50 µl drops of 2% ammonium molybdate (Sigma Aldrich, St. Louis, MO) for 2 min. After air-drying, grids were observed with a JEOL electron microscope JEM-1200EX (JEOL, Toyko, Japan).

### Mice housing and handling

For all experiments, 6 week-old female BALB/c mice were purchased from Charles River Breeding Laboratory Inc. (Wilmington, MA) and maintained in a biohazard animal facility. Anesthesia (isofluorane dispensed through a precision vaporizer) was used for blood collection from the retro-orbital plexus. All studies were approved by the Institutional Animal Care and Use Committee (IACUC) of the University of Maryland Baltimore School of Medicine, and conducted in accordance with NIH guidelines [23].

### Mice LD50

For assessment of virulence, the hog gastric mucin assay was used [26]. Mice were injected by the intraperitoneal route (i.p.) with increasing 10-fold dilutions of bacteria; bacteria were harvested from overnight Lennox plates and suspended in PBS mixed with 10% (wt/v) Difco hog gastric mucin (Becton-Dickinson, Sparks, MD) to a final volume of 0.5 ml per mouse. Groups of six mice per dose per strain were tested. Mice were observed twice daily for 3 days for mortality or any signs of significant morbidity (ruffled fur, weight loss of 20% or more, collapse, difficulty breathing or severe dehydration), and those showing the above signs were euthanized according to IACUC directives. 72 h post-challenge, surviving mice were euthanized using CO_2_ asphyxiation followed by cervical dislocation. LD_50_ values were calculated by logistic regression analysis.

### Mucosal immunization and challenge

Fresh vegetative cultures of CVD 1901, CVD 1901*D* or CVD 1901*K* were pelleted, washed with PBS, and brought to a final concentration of ∼10^11^ cfu/ml. 10-µl aliquots were applied intranasally on day 0, 14, and 28 to mice (5 µl/nostril, ∼10^9^ cfu per mouse; 10–15 mice per group). A group immunized with PBS served as a negative control. Blood samples were collected prior to and after immunization and sera were stored at −70°C. Mice were challenged on day 56 with 3.3×10^5^ cfu per mouse of wild-type ATCC 9150 *S.* Paratyphi A, freshly prepared as described above for the LD_50_ studies. Iron (8 µl of 5% ammonium iron (III) citrate to 1 ml of mucin) was added to the bacterial suspension to increase virulence [27]. Following the challenge, mice were monitored every 6 h for 72 h for mortality or any signs of significant morbidity.

### ELISA

Total IgG antibodies and IgG subclasses against *S.* Paratyphi A flagella were determined by ELISA as previously described [28]. Briefly, 96-well plates were coated with *S.* Paratyphi A flagella (5 µg/well) or LPS (10 µg/ml). Samples were diluted in 10% dried milk in PBS containing 0.05% Tween 20 (PBSTM) and tested in duplicates. Specific antibodies were detected using HRP-labeled goat anti-mouse IgG, IgG1 and IgG2 (KPL Inc. Gaithersburg, MD) diluted in PBSTM followed by TMB Microwell Peroxidase Substrate solution (KPL). Titers were calculated by interpolation in a standard curve as the inverse of the dilution that produces an OD value of 0.2 above the blank (ELISA units/ml).

### Bactericidal assay

Bactericidal activity was assessed by a complement-mediated lysis of *S.* Paratyphi A using sera from immunized mice. Fresh vegetative wild-type ATCC 9150 (flagellated) or 9150*K* (non-flagellated) bacterial suspensions (10^6^ cfu/ml) were mixed with 30% guinea pig complement (Sigma-Aldrich, St. Louis, MO), added (1∶1) to heat-inactivated (56°C, 20 min) serially-diluted mouse sera, and incubated for 1 h at 37°C. Following incubation, bacteria were counted by plating. End point titers were defined as the last dilution that induced a ≥50% reduction in the number of bacteria incubated with complement alone without addition of serum.

### Opsonophagocytosis

Antibody-mediated bacterial uptake by macrophages was measured by seeding J774A.1 cells into 24-well microdilution plates and growing in DMEM supplemented with 5% FCS at 37°C with 5% CO_2_ to a confluent layer (2×10^5^ cells/well). Fresh vegetative wild-type ATCC 9150 bacteria were incubated with heat inactivated mouse serum (10% in PBS) for 30 min on ice, then added to the cell monolayer at a ratio of 1∶1. Following centrifugation (100×*g*, 10 min), the microdilution plate containing the monolayer and opsonized bacteria was incubated at 37°C with 5% CO_2_ for 30–45 min. External bacteria were removed by replacing the media with fresh media containing 100 µg/ml gentamicin, incubating for 30–45 min, followed by three PBS washes. The cells were lysed with 0.5% Triton x-100 and internalized bacteria were counted by plating.

### Statistical analysis

LD_50_ was estimated by logistic regression analysis. Continuous variables were compared among the groups using Kruskal-Wallis analysis of variance. Proportions in two groups were compared using the Fisher's exact test. Two-sided *p*-values<0.05 and one-sided *p*-values<0.025 were considered statistically significant.

## Results

### Selection of a parental *S.* Paratyphi A strain for developing attenuated vaccines

**Figure 1:**
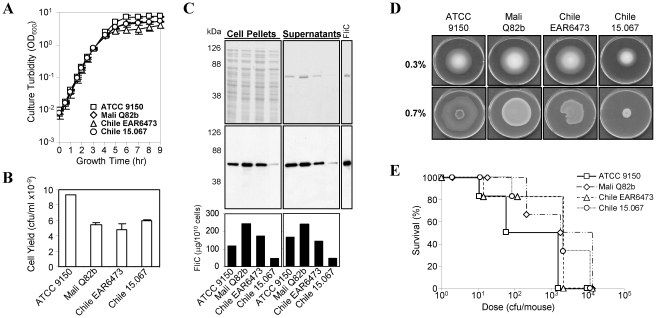
ATCC 9150 is highly culturable, expresses flagella moderately, and is highly virulent. (**A**) Growth curves of four *S.* Paratyphi A isolates cultured on Lennox medium at 37°C under high aeration (250 rpm, 1∶10 medium∶flask volume). (**B**) Final cell yields were recorded at time point of 8 h (t = 8) by performing live counts. Average yields from two independent experiments are shown. (**C**) Equivalent cell pellets and supernatants from t = 8 were analysed by SDS-PAGE and stained with Coomassie (upper panels) or subjected to Western blot analysis with anti-FliC antibody (middle), along with *S.* Paratyphi A FliC standard (100 µg). Estimation of FliC quantity was performed on the blots with Bio-Rad QuantityOne (lower). (**D**) Swim (0.3% agar) and swarm (0.7%) plates, following 8 h incubation at 37°C. (**E**) BALB/c mice were injected i.p. with 4 *S.* Paratyphi A isolates. Bacteria were collected from Lennox plates incubated at 37°C over-night, diluted in PBS, and mixed with hog gastric mucin. Groups of naïve BALB/c mice, 6 mice per group were challenged and monitored twice daily for three days. Data represent percent cumulative survival curves.

Four *S.* Paratyphi A wild-type strains were compared for growth rate, bacterial cell yield and ability to express FliC, including American Type Culture Collection strain ATCC 9150 and three clinical isolates Q82b (Mali), EAR6473 (Chile) and 15.067 (Chile). No significant differences in growth rate (Fig. 1A) or overall protein electrophoretic profiles (Fig. 1C) were observed among the strains, but ATCC 9150 consistently reached the highest cell yield when grown in rich liquid medium (Fig. 1B). FliC was a major component of the secretomes of all four strains (Fig. 1C, middle panel). The high amounts of flagellar protein found in the supernatants are a consequence of shear forces acting upon the cells during growth in shake flasks, causing filament shearing from the cell surface. Among the four strains, the Chilean isolate 15.067 expressed lower levels of FliC (Fig. 1C, lower panel).

To compare flagellar protein expression further, “swim” and “swarm” motilities were tested, each providing evidence of flagella functionality [29]. Swimming is assayed by growing the bacteria on semisolid medium (0.2–0.4% agar) where bacterial cells swim through water-filled channels in the agar, whereas swarming is observed following inoculation on the surface of solid medium (0.5–0.8% agar). Notably, swarming is associated with greater flagella expression than swimming [29]. In accordance with FliC expression (Fig. 1C), strain 15.067 had reduced motility (Fig. 1D).

We next tested whether variations of flagellar expression affect virulence in mice. Since *S.* Paratyphi A is avirulent in mice when administered orally or intranasally (i.n.), we adopted a mouse model used for *S.* Typhi (another human host-restricted pathogen) to determine LD_50_, in which bacteria are suspended in hog gastric mucin prior to intraperitoneal (i.p.) injection of BALB/c mice. This model has been used to assess the attenuation of candidate oral *S.* Typhi vaccines pre-clinically [26], [30] and reasonably predicted responses of humans given those strains in Phase 1 trials [31], [32]. Accordingly, young mice were injected i.p. with 10-fold dilutions of bacteria in 10% (w∶v) hog gastric mucin. ATCC 9150 was the most virulent, with an LD_50_ value of 52 cfu/mouse, while strains Q82b, EAR6473 and 15.067 exhibited LD_50_ values of 846, 199 and 692 cfu/mouse, respectively (Fig. 1E). ATCC 9150, with its excellent growth characteristics, copious flagella production and high virulence in mice, was therefore selected as the wild-type parent for construction of our vaccine candidates; the available genomic sequence of ATCC 9150 provided another rationale for using this strain [33].

### Deletion of *fliD* or *flgK* allows flagellin export

To export flagellin as monomers, we targeted two chromosomal loci, FliD (flagellar cap protein) and FlgK (a hook-filament junction protein), shown in *S.* Typhimurium to encode hook-associated proteins [17], [20], [34]. To assure the safety of our candidate strains, we first introduced a deletion in the chromosomal *guaBA* operon of ATCC 9150, which encodes two essential enzymes, inosine monophosphate dehydrogenase (GuaB) and guanine monphosphate synthetase (GuaA), involved in the *de novo* guanine nucleotide biosynthesis pathway. Resulting strain CVD 1901 was then further deleted for *fliD* (yielding CVD 1901*D*) or *flgK* (CVD 1901*K*). The *fliD* or *flgK* deletions were also introduced into ATCC 9150, leading to 9150*D* and 9150*K*.

**Figure 2:**
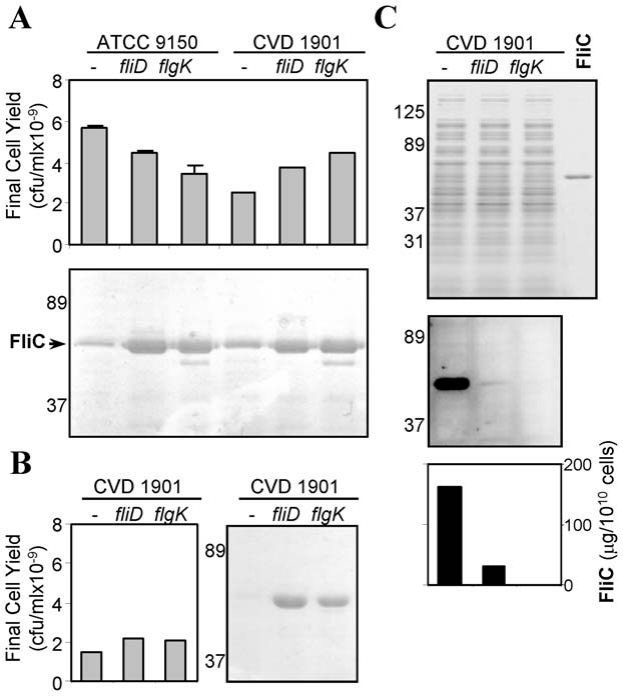
*S.* Paratyphi A Δ*fliD* and Δ*flgK* mutants shade FliC to culture supernatant. ATCC 9150 and five derivative mutants were grown on Lennox medium under high aeration. (**A**) Cell yields (**upper panel**) and Coomassie-stained SDS-PAGE (**lower**) of culture supernatants following 8 h of growth. (**B**) Cell yields and SDS-PAGE of supernatants from cultures grown over-night under low-aeration. (**C**) SDS-PAGE (**upper**), anti-FliC western blot (**middle**) and FliC quantitation (**lower**) of cell pellets from cultures grown over-night under low-aeration.

ATCC 9150 and the resulting five isogenic strains possessed indistinguishable growth rates when cultured under high aeration in appropriately supplemented rich liquid medium (data not shown). Thus, neither the *guaBA*, *fliD* or *flgK* mutations impaired growth, although bacterial cell yields were somewhat lower compared to ATCC 9150 (Fig. 2A top panel). As expected, analysis of secreted protein confirmed that higher FliC levels were found in supernatants of the Δ*fliD* and Δ*flgK* mutants compared to supernatants from their parental strains (Fig. 2A lower panel).

Differences in FliC expression between the *fliD* and *flgK* mutants and their parents were pronounced when bacteria were propagated as stationary broth cultures (low-aeration) where shear forces acting upon the cells are much lower. Under these conditions, no free FliC was observed in supernatants of either parental strain, yet FliC levels in supernatants of the *fliD* and *flgK* mutant cultures were as high as when grown under aerated conditions (shown for CVD 1901 and derived mutants, Fig. 2B). Immunoblotting with anti-FliC under low-aeration growth showed that the parental strain retained most flagella on the bacterial cells. In contrast, the mutants exhibited almost undetectable levels of FliC on the cell surface; CVD 1901*D* showed some residual FliC, while none was detected on the surface of CVD 1901*K* (Fig. 2C).

### *fliD* and *flgK* deletion mutants differ in export of flagellin monomers

**Figure 3:**
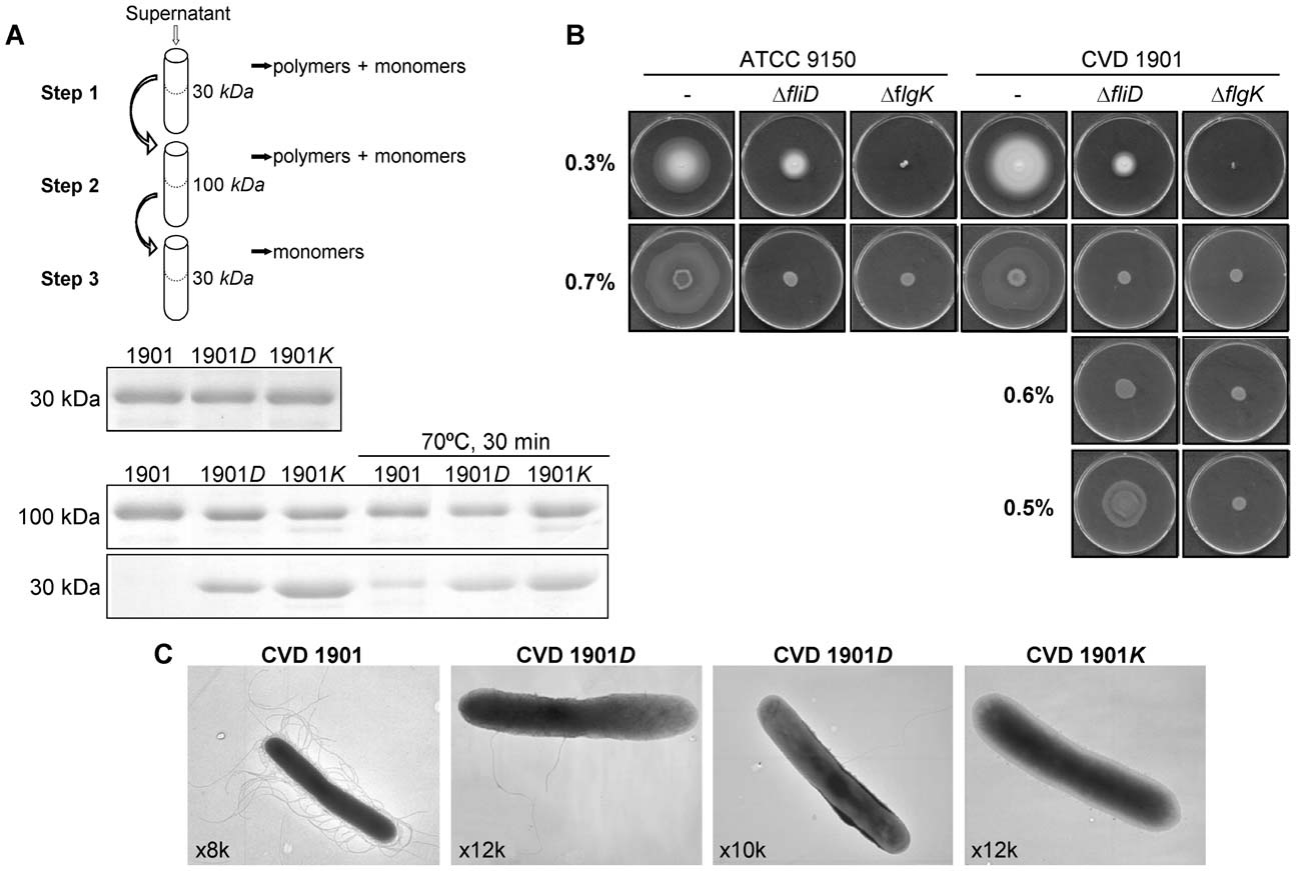
Δ*fliD* and Δ*flgK* mutants differ in their ability to export non-assembled flagellin. (**A**) Separation between monomeric and filamentous FliC using serial UF membranes. Supernatants were brought to equivalent FliC concentration using 30-kDa Amicon UF units and compared by a Coomassie-stained SDS-PAGE (upper gel). The concentrated samples, with or without heat treatment (70°C, 30 min), were then run through 100-kDa units and compared again (middle). Filtrates from the previous step were passed through 30-kDa units, and the final concentrates were detected (lower). (**B**) Swim (0.3% agar) and swarm (0.7, 0.6 and 0.5%) plates were scanned following 8 h incubation, 37°C. (**C**) EM images of negatively-stained bacteria, collected from swarm plates. For CVD1901*D*, cells carrying two and a single filament are shown. Numbers indicate the extent of magnification.

Export of FliC from the mutants was elucidated by a three-step characterization (Fig. 3A schema), using serial ultrafiltration (UF) membranes. First, conditioned media of CVD 1901, CVD 1901*D* and CVD 1901*K* cultures were adjusted to equivalent FliC concentrations using 30-kDa cutoff UF (Fig. 3A upper gel). Then, 100-kDa (Fig. 3A middle gel) followed by 30-kDa cutoff membranes allowed separation of monomeric from polymeric flagellin. Following the final 30-kDa passage, CVD 1901*K* supernatant contained the highest amount of flagellin, with less in the CVD 1901*D* supernatant and almost none in concentrate of the parental CVD 1901 strain (Fig. 3A lower). Thus, flagellin molecules in supernatants from cultures of the *fliD* and *flgK* mutants are in the unassembled form. However, in accordance with the results shown in Fig. 2C, some flagellin expressed by the CVD 1901*D* mutant is cell-associated, sheared off the cell surface during growth, and retained within the 100-kDa filter. As a control, all culture supernatants were heat-treated to dissociate the polymeric flagella and indeed some FliC was observed in the filtrate of the parental CVD 1901 strain following the 100-kDa UF (Fig. 3A middle and lower).

Second, comparing flagella functionality revealed identical swim and swarm diameters (% of plate) for ATCC 9150 versus CVD 1901; 39% versus 43% for swim and 45% versus 69% for swarm, respectively (Fig. 3B). Strains 9150*D* and CVD 1901*D* showed only swimming motility with a diameter of 11% and no swarming, while 9150*K* and CVD 1901*K* showed neither swim nor swarm motility. Decreasing the agar concentration in the swarming plates allowed swarming of the Δ*fliD* mutants, while the Δ*flgK* mutants remained non-motile (Fig. 3B). Finally, bacterial samples from the 0.7% swarm plates were examined by electron microscopy (EM). The negative stained bacterial cell images establish that the Δ*flgK* mutant is completely devoid of surface flagella, while the Δ*fliD* mutant carries one or two filaments (Fig. 3C).

### Δ*fliD* and Δ*flgK* mutants exhibit similar virulence to their parental strains

**Figure 4:**
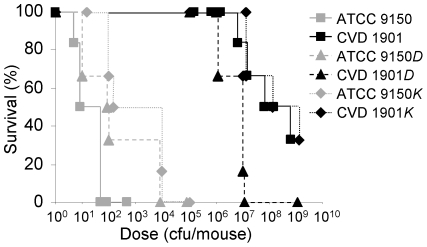
Δ*fliD* and Δ*flgK* mutations do not impair virulence of *S.* Paratyphi A in mice. Survival of naïve BALB/c mice infected with different strains of *S.* Paratyphi A at the indicated doses (cfu/mouse). Bacteria were collected from plates, diluted in PBS and mixed with hog gastric mucin. Groups of 6 mice were injected i.p. and monitored twice daily.

Virulence of the mutants was compared to wild-type ATCC 9150 by inoculating 6 week-old mice i.p. with bacteria suspended in hog gastric mucin. An LD_50_ value of 8.8 bacteria per mouse was calculated for the wild-type (Fig. 4). In contrast, CVD 1901 showed an LD_50_ of 3.0×10^7^ cfu/mouse. The LD_50_s for 9150*D*, 9150*K*, CVD 1901*D* and CVD 1901*K* were 17, 49, 3.4×10^6^ and 2.0×10^7^ cfu/mouse, respectively (Fig. 4). These results show clear attenuation only for strains harboring the *guaBA* deletion. Deletion of either *fliD* or *flgK* from ATCC 9150 attenuated the resulting strain by only half a log and did not work synergistically with the *guaBA* deletion. Whereas deletion of *flgK* or *fliD* in *S.* Paratyphi A did not alter bacterial virulence in this model, these mutations may nevertheless influence the protection conferred by vaccine strains also deleted in *guaBA*. Accordingly, we examined the ability of these mutants to protect against a *S.* Paratyphi A lethal challenge.

### A live attenuated strain provides superior protection if it has attached flagella rather than exporting flagellin monomers

Mice were immunized i.n. with ∼1×10^9^ cfu of CVD 1901, CVD 1901*D* or CVD 1901*K* on days 0, 14 and 28. Control mice received PBS. The i.n. route was chosen based on the robust immunity [35], [36] and protection [37] induced by attenuated *S*. Typhi administered by this route. Three weeks after the last immunization, mice were challenged with a lethal dose of wild-type ATCC 9150 (3.3×10^5^ cfu/mouse plus iron, see Materials and Methods). All control mice succumbed within 24 hours post-challenge (Table 3). The vaccine strains differed in their protective capacity, with CVD 1901 conferring significantly superior protection compared to CVD 1901*D* or 1901*K*.

**Table 3:** Recorded deaths within immunized mice challenge with a lethal dose* of *S.* Paratyphi A ATCC 9150 wild-type strain.

immunization	Mortality	Vaccine efficacy	*p* value***
PBS	8/8	-	-
CVD 1901	1/10^a^	90%	0.0002
1901*D*	8/15^b^	47%	0.026
1901*K*	7/10^c^	30%	0.15
Naïve**	0/5	-	-

*: Challenge dose for 52–54 week old mice was 3.3×10^5^ cfu, 0.125 µM Fe^+2^ in 10% hog gastric mucin.

**: Challenged with mucin but no bacteria.

***: Compared to PBS, from one-sided Fisher's exact test.

^a^ *vs* ^b^, *p* = 0.034 and ^a^ *vs* ^c^, *p* = 0.010, one-sided Fisher's exact test.

**Figure 5:**
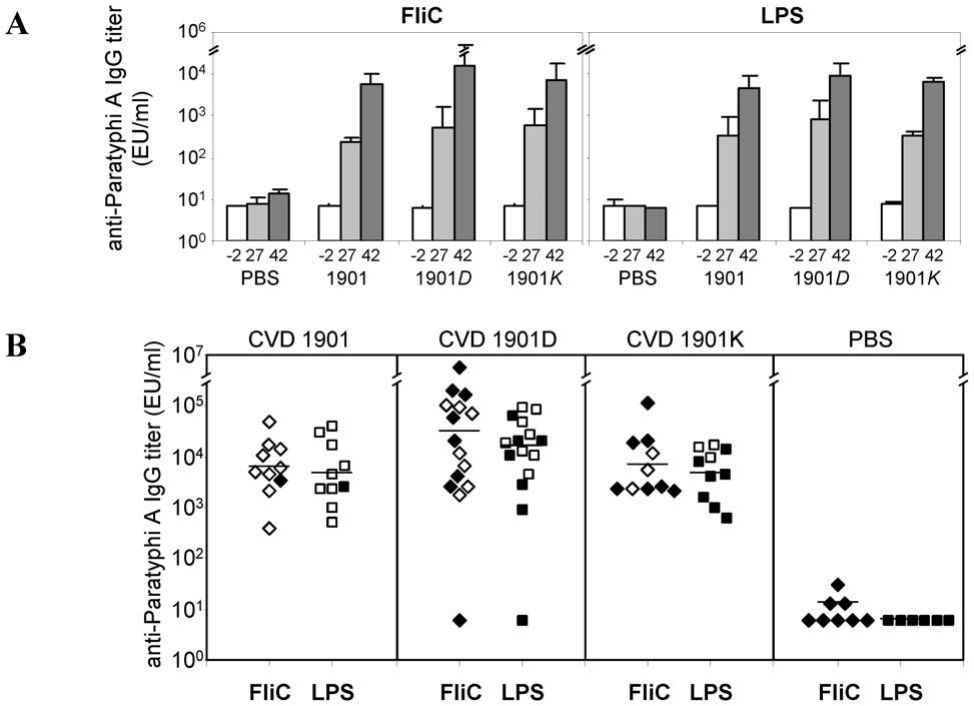
Serum IgG against *S.* Paratyphi A FliC and LPS do not correlate with protection. (**A**) Total serum anti-FliC and anti-LPS ELISA antibody titers (geometric means ± standard error of the mean) from days −2 (prior to vaccination), 27 (two weeks following first boost) and 42 (two weeks following second boost). *S.* Paratyphi A FliC was extracted from strain CVD 1902. LPS was extracted from strain CVD 1901. (**B**) Comparison of anti-FliC and anti-LPS total serum IgG titers at day 42 from individual mice. Closed shapes represent mice that succumbed to the challenge.

Serum IgG antibodies against FliC and LPS rose progressively after each immunization (Fig. 5A), reaching similar levels for all strains. Unlike CVD 1901 (which expresses many) and CVD 1901*D* (expresses a few) surface-associated flagella, CVD 1901*K* is devoid of flagella at the time of administration. Hence, FliC antibodies induced by CVD 1901*K* represent responses to *de novo* FliC synthesized *in vivo*, rather than antigen present at the time of immunization. The slightly higher titers detected in the mice immunized with CVD 1901*D* may reflect the combined effect of surface-associated and secreted FliC. Overall, there was no significant correlation between anti-FliC or anti-LPS antibody titers and survival (Fig. 5B), nor a correlation of survival with antibodies to *S.* Paratyphi A outer membrane protein fractions (data not shown). Thus, no serum IgG responses against major *Salmonella* surface antigens correlated with protection in this model.

### CVD 1901, CVD 1901*D* and CVD 1901*K* induce similar levels of anti-FliC IgG2a, whereas the flagellin-exporting mutants induce higher levels of anti-FliC IgG1

**Figure 6:**
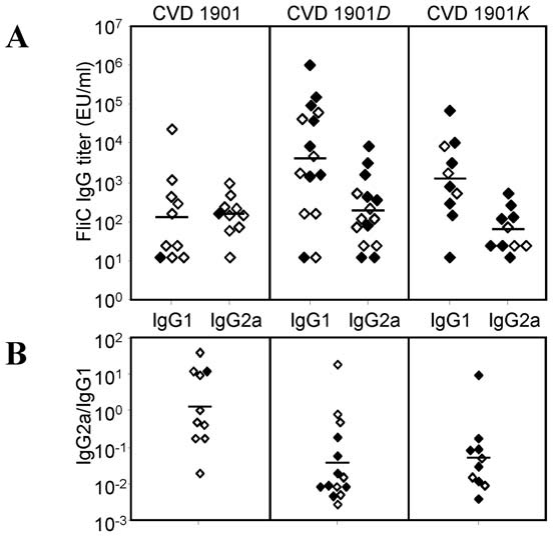
Immunization with CVD 1901*D* and CVD 1901*K* but not CVD 1901, induces high anti-FliC IgG1. Subclass distribution of IgG antibodies against *S.* Paratyphi A flagella. (**A**) Anti-FliC IgG1 and IgG2a titers detected in day 42 sera. (**B**) The ratio between the antibody subclasses. For (**A**) and (**B**), closed shapes represent mice that succumbed to the challenge.

Since the overall level of anti-FliC IgG among the immunized groups did not correlate with efficacy, we examined IgG antibody subtypes. While all three vaccine strains induced similar levels of anti-FliC IgG2a antibody, CVD 1901*D* and CVD 1901*K* induced strikingly high levels of anti-FliC IgG1, which were 50- and 10-fold higher than those induced by CVD 1901, respectively (Fig. 6A, *p* = 0.012). The IgG2a∶IgG1 geometric mean ratios were 1.2, 0.036, and 0.051 for CVD 1901, CVD 1901*D*, and CVD 1901*K* respectively, implying that a functional IgG2a-biased response, rather than elevated (and likely competing) IgG1 antibodies, might correlate with enhanced protection (Fig. 6B; *p* = 0.042).

### Cell-associated flagella of CVD 1901 elicit antibodies with opsonophagocytic rather than complement-mediated bactericidal activity

Noting the differences in anti-FliC IgG subtype antibody responses induced by the different live vaccines, we next studied functional activity of the antibodies. Antibody switching to different IgG subclasses requires T-cell help (T_H_) during antigen priming; the presence of IgG1 reflects T_H_2 subset activity, whereas IgG2a indicates a T_H_1-type response. Since live vaccine carrying cell-associated FliC exhibited higher potency compared with flagellin-secreting strains, we further examined the potential contribution of the T_H_1-associated antibody response induced by these strains in protection against *S*. Paratyphi A challenge. The T_H_1 subset is responsible for many cell-mediated functions and favors the production of IgG2a antibodies with opsonophagocytic capacity that bind to high-affinity Fc receptors on macrophages [38]. These antibodies activate the complement system more readily than IgG1 antibodies [39] and efficiently mediate antibody-dependent cell-mediated cytotoxicity [40].

**Figure 7:**
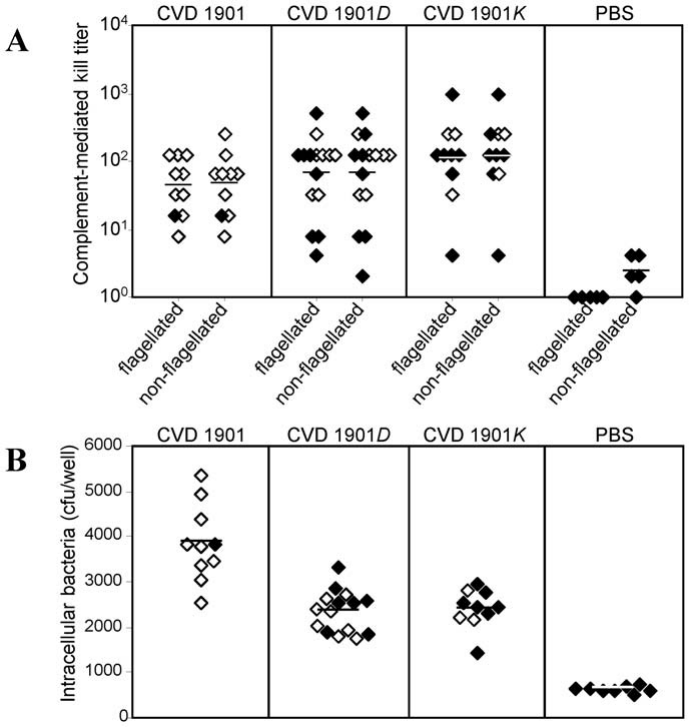
CVD 1901 cell-associated flagella elicits antibodies with opsonophagocytic rather than complement-mediated bactericidal activity. (**A**) Serum-induced killing of ATCC 9150 (flagellated) and 9150*K* (non-flagellated) bacteria mediated by complement. Sera are from day 42 of the experiment described in Fig. 5. (**B**) Opsonophagocytic activity of sera withdrawn at day 27 in J774A.1 macrophage against the wild-type ATCC 9150 strain. For (**A**) and (**B**), closed shapes represent mice that succumbed to the challenge.

Complement-mediated antibody killing (bactericidal) of wild-type ATCC 9150 was assessed by incubating bacteria with serial dilutions of heat-inactivated sera from immunized mice to which guinea pig complement was added. Sera from naïve mice established the background activity. For the three sera sets, analogous bactericidal titers against ATCC 9150 were detected with no significant differences between the groups (Fig. 7A, *p* = 0.28). Similar results were obtained when the assay was repeated with the non-flagellated 9150*K* strain (Fig. 7A, *p* = 0.22), indicating that anti-FliC antibodies do not play a major role of *S.* Paratyphi A complement-mediated killing in this mouse model.

We next examined opsonophagocytic activity using a macrophage culture assay that probes the ability of the sera to facilitate uptake of ATCC 9150. Average numbers of intracellular bacteria of 3862, 2383 and 2131 per 5×10^6^ bacteria per well were recovered for sera from mice immunized with CVD 1901, CVD 1901*D*, and CVD 1901*K*, respectively (Fig. 7B), indicating a clear increased uptake for CVD 1901 sera (*p* = 0.0002). These data suggest enhanced anti-FliC antibody-mediated clearance of the organism by phagocytic cells induced by vaccines expressing cell-associated rather than exported FliC, which might be contributing to the vaccine-induced survival from *S.* Paratyphi A challenge *in vivo*.

## Discussion

Flagellar protein is highly immunogenic and immunomodulatory via stimulation of TLR5, yet questions remain over its role in mediating protection against *Salmonella* [41]–[45]. Whereas purified Phase 1 flagella filaments or FliC subunits from *S.* Typhimurium [46] or *S.* Paratyphi A [10] inoculated parenterally protect mice against parenteral challenge with wild-type *Salmonella* of the homologous serovar, equipoise exists over whether flagellar protein contributes to protection when presented by live mucosal or parenteral inactivated whole cell vaccines. Flagellin expression is not needed for live oral *S.* Typhimurium vaccines to protect against wild-type challenge [47], while human studies indicate an important role for cell-associated flagella in the protection conferred by parenteral inactivated whole cell typhoid vaccines. Inactivated whole cell vaccines (most derived from wild-type strain Ty2) that provided superior protection also elicited higher anti-flagellar antibodies [48], [49]. Importantly, no efficacy was observed in a large-scale controlled field trial when the inactivated vaccine was based on non-flagellated *S.* Typhi mutant TNM1, derived from strain Ty2 [50], suggesting that inactivated whole cell vaccines must express flagella in order to protect humans [50].

We employed attenuated *S.* Paratyphi A to investigate the protective capacity of the flagellar subunit protein FliC expressed by live mucosal vaccines. Since mucosally-administered live vaccines assure *in vivo* expression and presentation of flagellar antigens in a native form, we engineered *S.* Paratyphi A ATCC 9150 with specific deletions affecting flagella filament biosynthesis. SDS-PAGE and western blotting (Fig. 2A–C), UF fractionation (Fig. 3A), motility assays (Fig. 3B) and EM (Fig. 3C) established the phenotypes of these FliC-exporting mutants. While ATCC 9150 and CVD 1901 almost exclusively produce flagellar protein as polymer filaments, 9150*D* and CVD 1901*D* (lacking flagellum cap protein) express only one or two filaments. In contrast, 9150*K* and CVD 1901*K* (lacking a flagellar hook-associated protein) are completely devoid of flagella. Studies with *S.* Typhimurium explain why *fliD* mutants carry a few intact flagellum polymers. In Δ*fliD S.* Typhimurium mutants [17], the tips of the hooks are intact and serve as effective heteronuclei for soluble FliC units to re-associate and form a functional flagellum. A filament already initiated has higher affinity for the newly added monomers, which elongate the single filament rather than form more short filaments.

Our engineered strains mutated in *fliD* or *flgK* but unaltered in growth characteristics or virulence (Fig. 4) provided a uniform background to explore the contribution of flagellar protein to immunity and protection. Mucosal immunization of mice with the live vaccines followed by subsequent i.p. challenge with virulent *S.* Paratyphi A showed prominent differences in the level of protection conferred in the face of a potent challenge that killed 100% of control mice (Table 3). CVD 1901 carrying many intact cell-attached flagella conferred 90% vaccine efficacy (*p* = 0.0002), while CVD 1901*D* with just a few cell-attached flagella provided 47% efficacy (*p* = 0.026); CVD 1901*K* with no attached flagella elicited only 30% efficacy (*p* = 0.15). Mortality was significantly lower in CVD 1901 recipients (1/10 mice) than in mice immunized with CVD 1901*K* (7/10 mice, *p* = 0.01) or CVD 1901*D* (8/15 mice, *p* = 0.04). These data indicate an advantage for live mucosal *Salmonella* vaccines having cell-attached FliC filaments.

The serum anti-FliC IgG titer did not correlate with protection. In fact, the less protective live vaccines that export flagellin subunits actually stimulated slightly higher anti-FliC IgG antibody titers (Fig. 5A–B). However, when we dissected the anti-FliC response we found that while all three vaccine strains induced IgG2a anti-FliC, the flagellin-secreting strains (CVD 1901*D* and 1901*K*) induced significantly higher IgG1 anti-FliC titers (Fig. 6, *p* = 0.012). IgG1 or IgG2a serum antibody responses in mice imply induction of T_H_2- or T_H_1-type subsets, respectively. Thus, both soluble and cell-attached polymeric FliC evoke T_H_1-directed switching to IgG2a but the IgG1 response is related to context. Whereas strong serum IgG1 anti-FliC responses were elicited by soluble exported FliC, this protein did not induce a strong IgG1-dependent T_H_2 response when presented as a bacterial cell-attached polymer, as observed for CVD 1901. Others have reported that mucosal administration of purified *S.* Typhimurium flagellin elicits a strong T_H_2-type response [51]–[53], while attached flagella on *S.* Typhimurium induce predominantly a T_H_1-dependent response [53]. Thus, the type of response against *S.* Typhimurium FliC did not seem to be determined by any intrinsic properties of FliC but rather appeared to be influenced by the form in which FliC was encountered, either as a soluble or cell-associated antigen [53]. Both monomeric and polymeric FliC induced T_H_2 responses provided these proteins were intact and not attached to cells, and only FliC on cells induced mainly a T_H_1 response [53]. Similar observations have been shown for *Lactobacillus*-associated and soluble FliC [54], indicating a general mechanism for anti-FliC antibody switching. We document the identical behavior for *S.* Paratyphi A FliC protein. However, our study advances the field since, in contrast to earlier reports, we presented FliC *in vivo* as attached whole flagella or as FliC monomers exported by live bacteria; previously, flagellin monomers or polymers were administered as purified protein.

Although many studies have described the immune responses to FliC, we report an association between a specific IgG subtype and protection. Vaccination with monomeric flagellin-exporting live vaccines induced stronger IgG1 anti-FliC responses but less protection against challenge with virulent *S.* Paratyphi A, suggesting that a pronounced T_H_2 response does not predict functional immunity.

One must ponder why certain specific antibodies elicited following vaccination with whole organisms fail to protect. One possibility is that some antibodies in excess may compete or block specific phagocyte Fc receptors that endocytose or phagocytose antibody-coated microorganisms [55]. Overwhelming Fc receptors may interfere with clearance of the pathogen. An excess of IgG1 anti-FliC antibodies induced by the exported FliC may prevent binding of the IgG2a subtype anti-FliC that is critical for clearing *S.* Paratyphi A infection. Alternatively, certain antibodies that lack relevant biological activity may actually enhance rather than control infection; thus, the high IgG1 anti-FliC may enhance the uptake of bacteria into cells without triggering killing. Antibodies that engage Fc receptors and enhance infection have long been known for *Chlamydia trachomatis* [56].

The T_H_1 subset is responsible for cell-mediated functions such as activation of cytotoxic T cells and production of opsonization-promoting IgG antibodies that bind to high-affinity Fc receptors and interact with the complement system. T_H_1 cells produce IL-2 and IFN-γ that promote the differentiation of fully cytotoxic T_C_ cells, which are suited to respond to intracellular pathogens. IFN-γ is a defining cytokine of the T_H_1 subset and activates macrophages to increase microbicidal activity [57]. IFN-γ secretion by T_H_1 cells also induces antibody class switching to IgG classes (IgG2a in the mouse) that support phagocytosis and complement fixation. Finally, in immunized mice most *Salmonella*-specific cells secreting IFN-γ are also FliC-specific [46].

Complement-dependent serum bactericidal antibodies [SBA] are one functional serological response in humans exposed to *Salmonella* pathogens or vaccines [58]. Our results show that SBA did not correlate with protection (Fig. 7A). Replacing the flagellated WT *S.* Paratyphi A ATCC 9150 utilized in the SBA assay with non-flagellated strain 9150*K*, did not alter SBA titers, indicating that anti-FliC antibodies did not function in this assay or contributed only inconsequentially. SBA must bind antigenic determinants on or very near the bacterial surface so that complement may interact with lipid membrane components. Thus, antibodies directed against flagella may be ineffective as SBA.

By contrast, we found antibodies mediating opsonophagocytic activity to be a useful functional correlate of protection against *S.* Paratyphi A (Fig. 7B), as has also been proposed for invasive non-typhoidal *Salmonella* infections [59]. A difference was observed in the opsonophagocytic activity of sera collected from mice immunized with CVD 1901 versus sera from mice immunized with CVD 1901*D* or 1901*K*. Sera from CVD 1901 mice readily mediated uptake of virulent *S.* Paratyphi A by phagocytes.

For every human vaccine for which a correlate of immunity exists [60], the correlate is a serum antibody. Live *Salmonella* vaccines elicit both cell-mediated and antibody responses and our studies shed light on the role of immune responses to flagellar protein elicited by a rationally-attenuated *S.* Paratyphi A live vaccine. In addition, the unpolymerized FliC monomers produced by our engineered strains could be exploited as subunit vaccines or as a platform for engineered FliC-heterologous antigen fusions that might not otherwise be supported within the confines of a polymerized flagellum. An additional byproduct of our research is the demonstration of a small animal model that allows, following mucosal immunization, the efficacy of live *S.* Paratyphi A vaccines to be assessed, including discernment among various candidates.
