# Expression of Concern: Cell-Associated Flagella Enhance the Protection Conferred by Mucosally-Administered Attenuated *Salmonella* Paratyphi A Vaccines

**DOI:** 10.1371/journal.pntd.0012160

**Published:** 2024-05-06

**Authors:** The PLOS Neglected Tropical Diseases Editors

Following the publication of this article [1], concerns were raised about results reported in Figs. 1 and 3.

1) In Fig. 1C when levels are adjusted to visualize the background, there appears to be a vertical discontinuity between lanes 3 and 4 of the “Cell Pellets” western blot panel. Image data provided for this panel (S1 File) clarified that lanes were spliced out in preparing the figure. The lanes of this original blot that were removed in the final published figure represent FliC production from additional clinical isolates of *S*. Paratyphi A that were not discussed in [1].
2) In Fig. 3A:
  - In the upper 30-kDa panel, when levels are adjusted to visualize the background there appear to be vertical discontinuities after lanes 1 and 2.
  - In the 100-kDa panel, when levels are adjusted to visualize the background there appear to be vertical discontinuities after lanes 1, 3, and 4.
  - The bands in lane 1 of the 100-kDa panel and in lanes 1 and 3 of the upper 30-kDa panel appear similar.

The original, full-size gel images underlying these results are no longer available and so the issues cannot be fully resolved. However, the authors confirmed that the upper 30-kDa panel in the published figure is a composite of cropped Coomassie gel image fragments, and they provided lab book images in support of the Fig. 3A results.

The lab book image in S2 File supports the 100-kDa and lower 30-kDa panels. For the 100-kDa panel, the Δ*fliD* and Δ*flgK* bands on the lab book image appear to match the data in the published figure but the wt bands do not. In contrast to the published figure, the lab book gel image shows a markedly lower protein level in the heat treated wt (1901) lane compared to the other lanes.

The lab book image in S3 File shows full gel images from a replicate experiment, with results corresponding to all three gel panels of Fig. 3A minus the heat treatment condition. The results in this lab book image support those presented in the corresponding Fig. 3A panels.

Overall, the data shown in the lab book images support the claim made in the article for Fig. 3A, “Following the final 30-kDa passage, CVD 1901*K* supernatant contained the highest amount of flagellin, with less in the CVD 1901*D* supernatant and almost none in concentrate of the parental CVD 1901 strain (Fig. 3A lower).”

3) In Fig. 3B, the ATCC 9150 Δ*flgK* 0.7% and CVD 1901 Δ*flgK* 0.5% panels appear similar. The authors noted that the original images for this experiment are no longer available, but they provided results from a replicate experiment for the 0.3% and 0.4% agar conditions (S4 File) that support the conclusion that *fliD* and *flgK* mutants have less functional flagella than parental strains.

The *PLOS Neglected Tropical Diseases* Editors issue this Expression of Concern to notify readers of the above issues and provide the available data for the figures of concern.

## Supporting information

S1 File Image data to support Fig. 1C. (TIF)

S2 File Lab book image supporting the 100-kDa and lower 30-kDa panels of Fig. 3A. The authors stated that this shows intact gel images with lines overlaid for clarity of presentation.
(TIF)

S3 File Lab book image showing results of a replicate experiment supporting Fig. 3A. (TIF)

S4 File Replicate experiment (0.3% and 0.4% agar conditions) to support Fig. 3B. (TIF)
