# Supplementary figures and images for: Expression of Concern: Cell-Associated Flagella Enhance the Protection Conferred by Mucosally-Administered Attenuated Salmonella Paratyphi A Vaccines
Source: PLoS Negl Trop Dis. 2024 May 6;18(5):e0012160. doi: 10.1371/journal.pntd.0012160 (PMC11073665; doi:10.1371/journal.pntd.0012160)

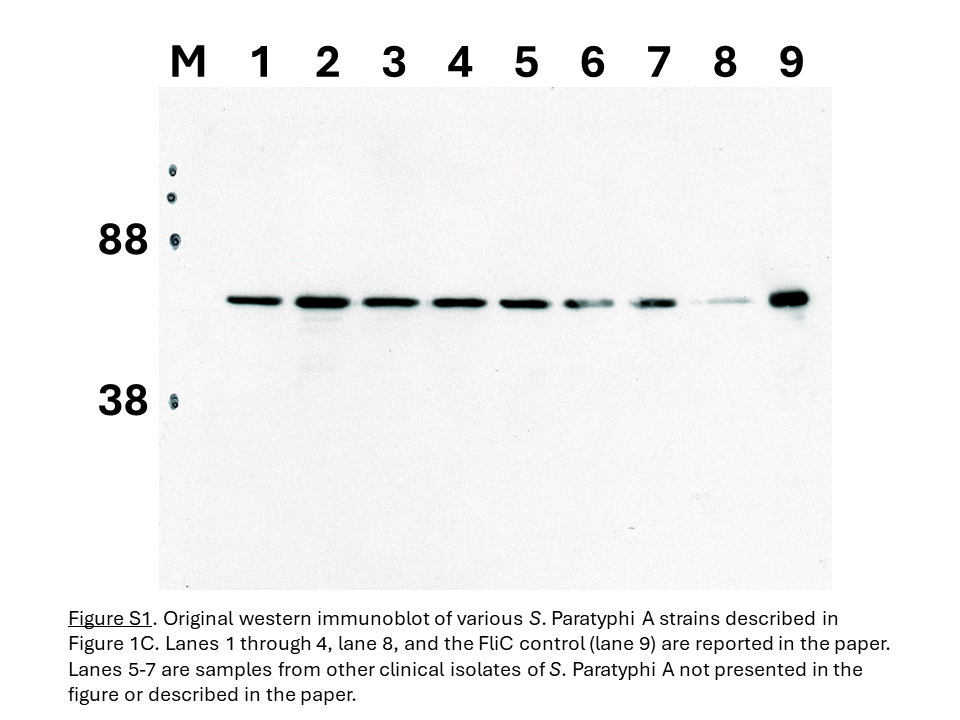

Supplement: S1 File — (TIF) [file pntd.0012160.s001.tif]

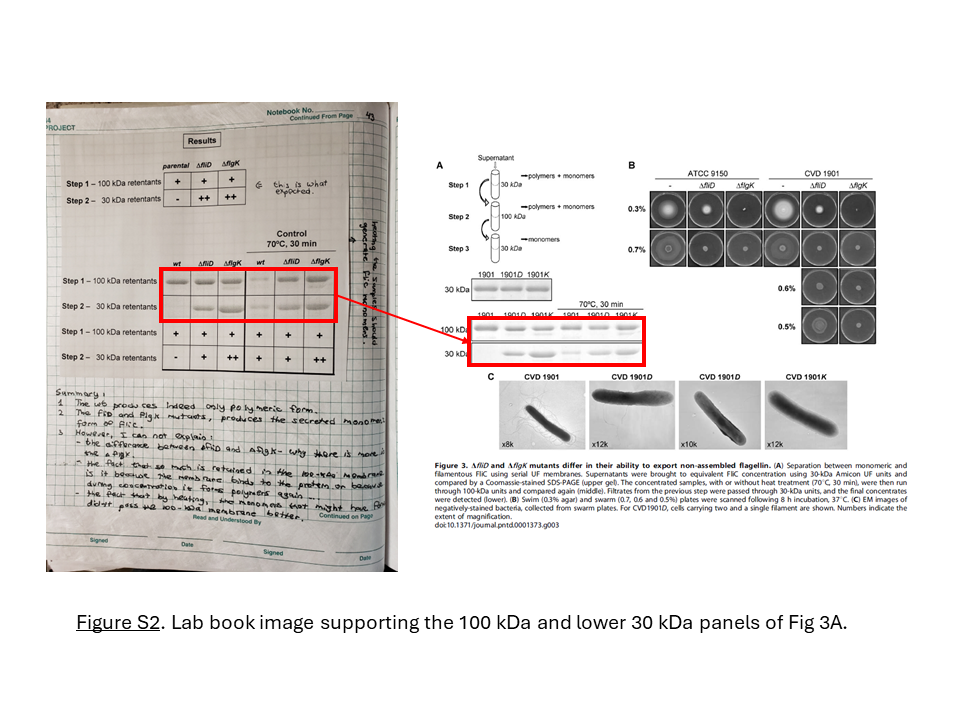

Supplement: S2 File — The authors stated that this shows intact gel images with lines overlaid for clarity of presentation. (TIF) [file pntd.0012160.s002.tif]

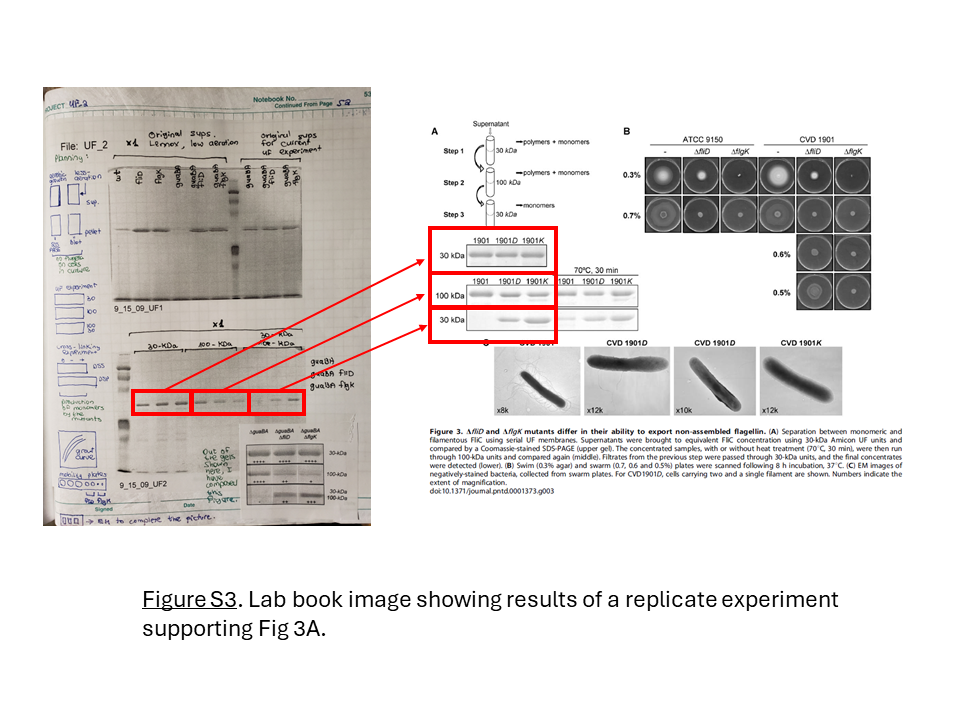

Supplement: S3 File — (TIF) [file pntd.0012160.s003.tif]

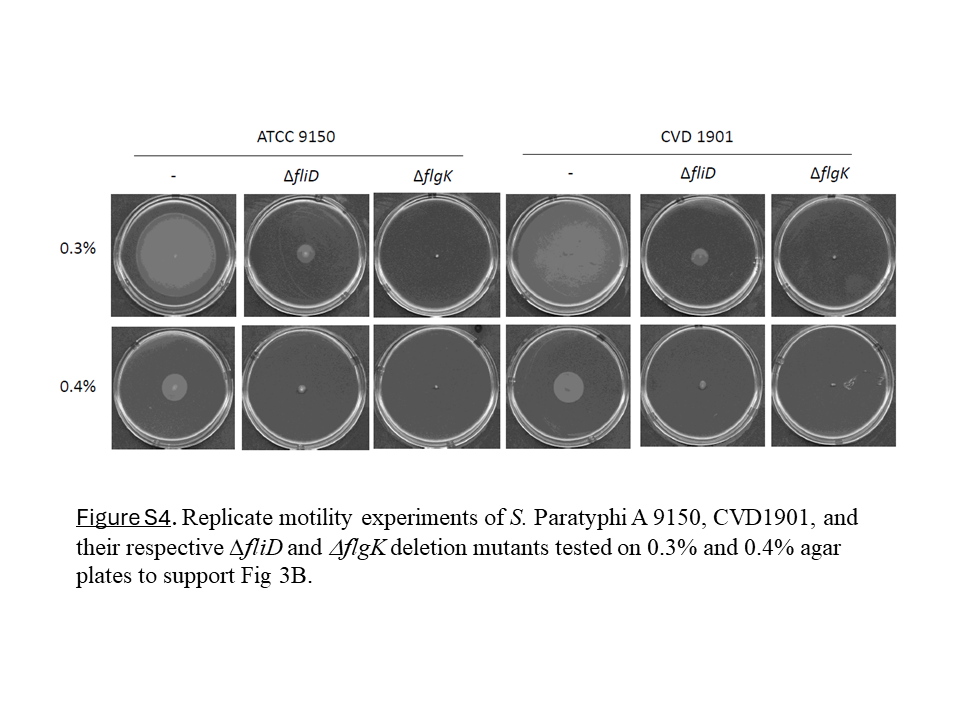

Supplement: S4 File — (TIF) [file pntd.0012160.s004.tif]
